# Single Nucleotide Polymorphisms in *IL1B* and the Risk of Acute Coronary Syndrome: A Danish Case-Cohort Study

**DOI:** 10.1371/journal.pone.0036829

**Published:** 2012-06-29

**Authors:** Jakob Gerhard Stegger, Erik Berg Schmidt, Anne Tjønneland, Tine Iskov Kopp, Thorkild I. A. Sørensen, Ulla Vogel, Kim Overvad

**Affiliations:** 1 Department of Cardiology, Center for Cardiovascular Research, Aalborg Hospital, Aarhus University Hospital, Aalborg, Denmark; 2 Danish Cancer Society Research Center, Copenhagen, Denmark; 3 National Food Institute, Technical University of Denmark, Soborg, Denmark; 4 Institute of Preventive Medicine, University of Copenhagen, Copenhagen, Denmark; 5 National Research Centre for the Working Environment, Copenhagen, Denmark; 6 Department of Epidemiology, School of Public Health, Aarhus University, Aarhus, Denmark; Innsbruck Medical University, Austria

## Abstract

**Background:**

Interleukin-1B (IL-1B) is a key pro-inflammatory cytokine that has been associated with the development of atherosclerosis and myocardial infarction. However, the prospective associations between functional single nucleotide polymorphisms (SNPs) in *IL1B* and incident acute coronary syndrome (ACS) have not been thoroughly investigated. The aims of this study were to examine the associations between individual SNPs in and SNP haplotypes of the promoter region of *IL1B* and incident ACS in a prospective study. Furthermore, we wanted to explore potential interactions with other risk factors for ACS on an additive scale.

**Methodology/Principal Findings:**

The present study was based on the Danish prospective study Diet, Cancer and Health comprising more than 57 000 participants aged 50–64 at baseline. During a median follow-up of 7.2 years we identified 989 cases of incident ACS (755 men and 234 women). All cases were validated by review of medical records, and information on covariates was collected by study technicians. The study was conducted according to a case-cohort study design including ACS cases and a sex-stratified sub cohort of 1663 participants drawn randomly from the entire cohort. Weighted Cox proportional hazard models with age as time axis were used in the statistical analyses. Individual *IL1B* SNPs, SNP haplotypes, or haplotype combinations were not significantly associated with incident ACS, and, likewise, we found no evidence of interaction on an additive scale between IL1B haplotypes and risk factors, respectively.

**Conclusions/Significance:**

Genetic variation in the promoter region of *IL1B* may not be associated with incident ACS in men or women above the age of 50 years.

## Introduction

Interleukin-1B (IL-1B) is a key pro-inflammatory cytokine that induces the production of other cytokines, adhesion molecules, and metalloproteinases [1], [2]. The IL-1 signaling pathway has been associated with the development of atherosclerosis in animal studies[3]–[5], and IL-1B mRNA is increased in human atherosclerotic arteries [6]. In a human case-control study, the -511T single nucleotide polymorphism (SNP) in *IL1B* was associated with a lower release of IL-1B from human mononuclear cells and a relatively low risk of myocardial infarction at young age [7]. Thus, *IL1B* expression by itself may influence the inflammatory processes in the arterial wall that lead to the development of atherosclerotic plaques and acute coronary syndrome(ACS)[8]–[10]. Furthermore, *IL1B* polymorphisms may interact with obesity, as the effect of obesity on plaque development is partly mediated by the release of inflammatory cytokines from adipose tissue compartments [11]. Likewise, the *IL1B* -3737T polymorphism modifies a binding site for the anti-inflammatory p50 subunit of the NF-kB transcription factor family [12], and thus there may be interaction between the *IL1B* -3737T genotype and the *NFKB1* -94 ATTG ins/del polymorphism which modifies the protein level of the p50 subunit and by itself is positively associated with the risk of ACS [13].

The entire *IL1B* gene has been sequenced, and several SNPs have been identified in the promoter region, but only four (T-31C, C-511T, G-1464C, and C-3737T) seem to be interesting regarding prevalence and functionality [12]. However, T-31C and C-511T are in complete linkage in Danes [14], leaving three functional polymorphisms in this population. The SNPs are all located in the same promoter region, and to address the combined effects of concurrent SNPs, we also performed analyses of functional SNP haplotypes as they exist in the human genome.

The aims of this study were to examine the primary associations between individual SNPs as well as SNP haplotypes and haplotype combinations in *IL1B* and incident ACS in a prospective study. Furthermore, we wanted to explore the potential interaction with obesity and the *NFKB1* -94 ATTG ins/del polymorphism, respectively. We hypothesized that SNPs and SNP haplotypes associated with higher levels of *IL-1B* expression would be positively associated with the incidence of ACS. Likewise, we hypothesized that the combined effect of SNP haplotypes associated with higher levels of *IL-1B* expression and obesity would be additive or greater.

## Materials and Methods

### Study Population

The present study was based on the Danish prospective study Diet, Cancer and Health, which has previously been described in detail [15]. In brief, from November 1993 to May 1997, all men and women aged 50–64 years, born in Denmark, and with no previous cancer diagnosis registered in the Danish Cancer Registry were invited to participate in the study. The Diet, Cancer and Health study was approved by the National Committee on Health Research Ethics (journal nr. (KF) 01-345/93) and the Danish Data Protection Agency. Written informed consent was obtained from all participants to search information from medical registers.

A total of 80 996 men and 79 729 women were invited to participate in Diet, Cancer and Health, and 27 178 men (34%) and 29 875 women (37%) consented to participate. During a median follow up of 7.2 years, 872 male and 272 female cases of incident ACS were diagnosed. To preserve biological material the present study was conducted as a case-cohort study and genotyping was only performed on cases and participants in a sex-stratified subcohort initially comprising 1869 participants drawn randomly from the entire cohort ultimo 2003.

We excluded 1011 participants with prevalent ACS, and 564 participants who, due to delay in the Danish Cancer Registry, were erroneously included in Diet, Cancer and Health, as they had been diagnosed with cancer before inclusion. Furthermore, we excluded 460 participants for whom information on exposure or confounder variables were missing. After exclusions there were 755 male and 234 female cases of incident ACS, and the subcohort consisted of 887 men and 776 women. The subcohort included 32 participants (26 men and 6 women) who later became cases.

### Genotyping

DNA was isolated from frozen lymphocytes as previously described [16]–[18]. The IL1B T-31C (rs1143627), IL1B G−1464C (rs1143623), and IL1B C−3737T (rs4848306) genotypes were determined on an ABI 7900HT using allelic discrimination (Applied Biosystems, Birkerød, Denmark) as previously described [14], [16].

Genotypes of NFKB1 ATTG ins/del (rs28362491) were determined by Taqman allelic discrimination (ABI 7500, Applied Biosystems) [19]. Controls with known genotypes were included in each run, and repeated genotyping of 10% of the samples yielded identical results. Cases and controls were mixed during genotyping and the case status of samples was blinded during genotyping.

### Endpoint

The endpoint was incident ACS (International Classification of Disease (ICD) 8: 410–410.99 or ICD 10: I20, I21.0–I21.9), and potential cases were identified by linkage with the Danish National Patient Registry and the Danish Causes of Death Registry using the Danish Civil Registration System in which every Danish person is identified by a unique 10-digit number. The Danish National Patient Registry [20] holds information on diagnoses and procedures (e.g. percutaneous coronary intervention) in relation to all hospital ward admissions since 1977, while all visits at outpatient clinics and emergency rooms have been registered since 1995. The Danish Causes of Death Registry includes all deaths since 1943. Patients were registered in both registries in accordance with ICD 8 until January 1^st^ 1995 and subsequently according to ICD 10.

Potential cases of incident ACS in Diet, Cancer and Health were validated by review of medical records and classified in accordance with the guidelines of the American Heart Association and the European Society of Cardiology for use in epidemiology [21]. Genetic or anthropometric data were not available at the time of case validation.

### Covariates

Anthropometric data were obtained by trained laboratory technicians. Height was measured to the nearest 0.5 cm with the participants standing without shoes. Weight was measured using a digital scale with the participants wearing light clothing and recorded to the nearest 0.1 kg. Waist circumference was recorded to the nearest 0.5 cm and measured at the narrowest part between the lower rib and the iliac crest. Information on other potential confounders was obtained through questionnaires covering socio-demographic factors, lifestyle, and general health including prevalent diseases and medications.

### Statistical Methods

In accordance with the case-cohort design and to allow for delayed entry of the participants, the hazard ratios (HR) of incident ACS were estimated by weighted Cox proportional hazards regression models with age as the underlying time variable [22], [23]. The observation time was censored by death from other causes than ACS, emigration, or study end. The proportionality assumptions of the Cox proportional hazards models were evaluated graphically using log-log plots.

The distribution of obesity (waist circumference (WC), hip circumference (HC), and body mass index (BMI)) was described in accordance with the WHO classification, but analyses reported in tabulations were based on three categories according to the distribution among cases. WC and HC were described by splines when included as covariates. We adjusted for potential confounding from educational level (basic school, higher education 1–3 years, or higher education >3 years), smoking status (non-smoker, current <15, or ≥15 g/d), alcohol intake (spline), hypertension (yes, no, or unknown), diabetes mellitus (yes, no, or unknown), hypercholesterolemia (yes, no, or unknown). For women we also included menopausal status (pre, post, or unknown). Hypertension, hypercholesterolemia, and diabetes mellitus were not included in analyses with anthropometric exposure variables, as they are intermediate variables in these analyses. The “unknown” category refers to participants who had provided information, but could not be defined as “yes” or “no”. All splines were centered restricted cubic splines with three knots placed as suggested by Harrel [24].

Potential biological interaction on an additive scale between SNP haplotypes and obesity (BMI, WC adjusted for HC, HC adjusted for WC) or the *NFKB1* -94 ATTG ins/del polymorphism was explored as the relative excess risk due to interaction (RERI) and calculated as suggested by Rothman [25].

**Table 1:** Baseline characteristics of the subcohort and cases of incident acute coronary syndrome.

	Men		Women	
	Subcohort	Cases	Subcohort	Cases
Variable	(*n* = 887)	(*n* = 755)	(*n* = 776)	(*n* = 234)
Age	56.0 (51.3;63.4)	57.9 (51.7;64.1)	55.8 (51.0;62.7)	59.9 (52.4;64.2)
Postmenopausal	n/a	n/a	58.1%	71.1%
Educational level				
Basic school	10.4%	14.8%	21.7%	31.5%
Higher education 1–3 years	13.4%	17.4%	31.2%	33.2%
Higher education >3 years	76.0%	67.8%	47.2%	35.3%
Smoking status				
Non-smoker	61.6%	40.7%	63.5%	41.3%
Less than 15 g/d	10.5%	12.5%	15.5%	24.3%
More than 15 g/d	28.0%	46.9%	21.0%	34.5%
Alcohol (g/day)	20.0 (3.1,61.6)	17.3 (2.0;61.2)	8.8 (1.0;34.4)	6.0 (0.7;32.6)
Hypertension^1^	13.40%	22.1%	16.0%	38.7%
Diabetes mellitus^1^	2.5%	5.3%	1.0%	5.1%
Hypercholesterolemia^1^	9.6%	12.3%	5.9%	17.5%
Body Mass Index (kg/m^2^)	26.4 (22.5;31.1)	27.0 (23.3;32.3)	24.6 (21.0;30.6)	26.3 (21.4;32.6)
BMI grouped according to WHO				
Underweight	0.3%	0.1%	1.3%	1.3%
Normalweight	33.8%	26.6%	53.0%	37.9%
Overweight	50.5%	51.1%	33.3%	38.3%
Obese	15.3%	22.1%	12.5%	22.6%
Waist circumference (cm)	95.0 (84.0;108.0)	97.0 (87.0;112.0)	80.0 (69.0;97.0)	86.0 (71.0,101.0)
Hip circumference (cm)	100.0 (92.5;108.0)	101.0 (93.0;110.0)	100.5 (92.0;113.0)	102.0 (91.0;114.0)
*NFKB1* 94ATTG ins/del polymorphism				
Wildtype/Wildtype	40% (358)	35% (265)	37% (291)	38% (90)
Wildtype/Variant	45% (403)	48% (360)	47% (364)	46% (108)
Variant/Variant	14% (126)	17% (130)	16% (121)	15% (36)

Medians with 10th and 90th percentiles in brackets for continuous variables. Percentages for discrete variables. Diet, Cancer and Health, Denmark, 1993–2003.

^1^^)^Self-reported.

**Table 2:** Display of the 4 haplotypes describing more than 99% of the genotype combinations.

	Haplotype			
Polymorphisms	1	2	3	4
* IL1B* C−3737T	**C** (wildtype)	**T** (variant)	**C** (wildtype)	**C** (wildtype)
* IL1B* G−1464C	**G** (wildtype)	**G** (wildtype)	**C** (variant)	**G** (wildtype)
* IL1B* T-31C	**T** (wildtype)	**T** (wildtype)	**C** (variant)	**C** (variant)
*IL1B* promotor activity (Chen et al^1^ *)*	Low	Medium	Medium	High

Diet, Cancer and Health, Denmark, 1993–2003.

^1^^)^Chen H et al. Single nucleotide polymorphisms in the human interleukin-1B gene affect transcription according to haplotype context. Hum Mol Genet 2006 Feb 15;15(4):519–529.

**Table 3:** Cox proportional hazard ratios (HR) with 95% confidence interval in brackets for the association between single nucleotide polymorphisms in IL1B and incident acute coronary syndrome.

	MEN				WOMEN				ALL	
	*n*		Crude	Adjusted^1^	*n*		Crude	Adjusted^1^	Crude^2^	Adjusted^2^
Polymorphism	Subcohort	Cases	HR	HR	Subcohort	Cases	HR	HR	HR	HR
*IL1B* C−3737T										
Wildtype/Wildtype	31% (279)	31% (233)	1 (ref.)	1 (ref.)	33% (259)	29% (69)	1 (ref.)	1 (ref.)	1 (ref.)	1 (ref.)
Wildtype/Variant	49% (437)	48% (359)	0.96 (0.77;1.21)	0.90 (0.70;1.16)	47% (365)	53% (125)	1.24 (0.88;1.76)	1.24 (0.82;1.89)	1.02 (0.84;1.24)	0.96 (0.78;1.19)
Variant/Variant	19% (171)	22% (163)	1.09 (0.82;1.45)	1.04 (0.76;1.42)	20% (152)	17% (40)	1.00 (0.64;1.58)	1.11 (0.67;1.84)	1.07 (0.84;1.37)	1.03 (0.79;1.35)
Dominant variant model	–	–	1.00 (0.81;1.24)	0.94 (0.74;1.19)	–	–	1.17 (0.84;1.63)	1.21 (0.82;1.78)	1.04 (0.87;1.24)	0.98 (0.81;1.20)
Minor allele frequency	44%	–	–	–	43%	–	–	–	–	–
*IL1B* G−1464C										
Wildtype/Wildtype	54% (477)	53% (399)	1 (ref.)	1 (ref.)	52% (401)	53% (124)	1 (ref.)	1 (ref.)	1 (ref.)	1 (ref.)
Wildtype/Variant	39% (348)	40% (299)	1.03 (0.84;1.27)	1.04 (0.83;1.31)	40% (310)	41% (97)	1.01 (0.73;1.39)	0.86 (0.59;1.24)	1.03 (0.86;1.23)	1.00 (0.82;1.22)
Variant/Variant	7% (62)	8% (57)	1.13 (0.77;1.68)	1.10 (0.71;1.69)	8% (65)	6% (13)	0.73 (0.38;1.39)	0.69 (0.33;1.44)	1.03 (0.74;1.43)	1.02 (0.71;1.47)
Dominant variant model	–	–	1.05 (0.86;1.28)	1.05 (0.84;1.30)	–	–	0.97 (0.71;1.31)	0.83 (0.58;1.19)	1.03 (0.87;1.22)	1.00 (0.83;1.21)
Minor allele frequency	27%	–	–	–	28%	–	–	–	–	–
*IL1B* T–31C										
Wildtype/Wildtype	45% (397)	47% (352)	1 (ref.)	1 (ref.)	44% (341)	44% (104)	1 (ref.)	1 (ref.)	1 (ref.)	1 (ref.)
Wildtype/Variant	44% (390)	43% (321)	0.94 (0.76;1.16)	0.95 (0.75;1.19)	45% (345)	47% (109)	1.00 (0.73;1.39)	0.83 (0.57;1.20)	0.96 (0.80;1.14)	0.92 (0.76;1.12)
Variant/Variant	11% (100)	11% (82)	1.02 (0.73;1.42)	1.04 (0.73;1.50)	12% (90)	9% (21)	0.87 (0.50;1.50)	0.80 (0.44;1.48)	0.98 (0.74;1.31)	1.01 (0.74;1.38)
Dominant variant model	–	–	0.96 (0.78;1.17)	0.96 (0.77;1.20)	–	–	0.98 (0.72;1.33)	0.82 (0.58;1.17)	0.96 (0.81;1.14)	0.94 (0.78;1.13)
Minor allele frequency	33%	–	–	–	34%	–	–	–	–	–

Median follow up 7.2 years. Diet, Cancer and Health, Denmark, 1993–2003.

^1^^)^Age used as time axis. Adjusted for educational level, waist circumference, smoking status, alcohol consumption, hypertension, diabetes mellitus and hypercholesterolemia. Women also adjusted for menopausal status.

^2^^)^Also adjusted for sex.

**Table 4:** Cox proportional hazard ratios (HR) with 95% confidence interval in brackets for the association between haplotypes of single nucleotide polymorphisms in IL1B and incident acute coronary syndrome.

	MEN				WOMEN						ALL		
	*n*		Crude	Adjusted^1^	*n*			Crude	Adjusted^1^		Crude^2^		Adjusted^2^
Haplotypes (-3737; -1464; -31)	Subcohort	Cases	HR	HR	Subcohort		Cases	HR	HR		HR		HR
1 (wildtype; wildtype; wildtype)													
0 copies	60% (518)	61% (457)	1 (ref.)	1 (ref.)	59% (458)		58% (135)	1 (ref.)	1 (ref.)		1 (ref.)		1 (ref.)
1 copy	34% (291)	34% (256)	0.99 (0.80;1.22)	0.99 (0.78;1.25)	35% (268)		37% (86)	1.04 (0.75;1.44)	1.08 (0.75;1.57)		1.00 (0.84;1.20)		1.02 (0.84;1.25)
2 copies	6% (52)	6% (42)	0.92 (0.60,1.42)	1.06 (0.66;1.69)	6% (44)		6% (13)	0.96 (0.48;1.90)	1.11 (0.50;2.44)		0.93 (0.65;1.35)		1.06 (0.71;1.59)
1 or 2 copies (dominant model)	–	–	0.98 (0.80;1.20)	1.00 (0.80;1.24)	–		–	1.03 (0.75;1.41)	1.09 (0.76;1.56)		0.99 (0.83;1.18)		1.03 (0.85;1.24)
2 (variant; wildtype; wildtype)													
0 copies	31% (271)	31% (233)	1 (ref.)	1 (ref.)	34% (260)		29% (69)	1 (ref.)	1 (ref.)		1 (ref.)		1 (ref.)
1 copy	49% (423)	48% (359)	0.97 (0.77;1.22)	0.93 (0.72;1.19)	47% (361)		53% (125)	1.25 (0.88;1.77)	1.26 (0.83;1.90)		1.03 (0.85;1.25)		0.98 (0.79;1.21)
2 copies	19% (167)	22% (163)	1.10 (0.83,1.46)	1.06 (0.77;1.44)	19%(149)		17% (40)	1.00 (0.64;1.58)	1.12 (0.67;1.85)		1.08 (0.85;1.37)		1.05 (0.80;1.36)
1 or 2 copies (dominant model)	–	–	1.01 (0.81;1.25)	0.96 (0.76;1.22)	–		–	1.18 (0.84;1.64)	1.22 (0.83;1.80)		1.04 (0.87;1.25)		1.00 (0.82;1.22)
3 (wildtype; variant; variant)													
0 copies	54% (467)	53% (399)	1 (ref.)	1 (ref.)	52% (397)	53% (124)		1 (ref.)	1 (ref.)	1 (ref.)			1 (ref.)
1 copy	39% (334)	40% (299)	1.04 (0.85;1.28)	1.06 (0.84;1.33)	40% (308)	41% (97)		1.01 (0.74;1.40)	0.87 (0.60;1.25)	1.04 (0.87;1.24)			1.02 (0.84;1.24)
2 copies	7% (60)	8% (57)	1.14 (0.77,1.68)	1.11 (0.72;1.71)	8% (65)	6% (13)		0.73 (0.38;1.39)	0.69 (0.33;1.44)	1.03 (0.74;1.44)			1.03 (0.71;1.48)
1 or 2 copies (dominant model)	–	–	1.06 (0.87;1.29)	1.07 (0.86;1.33)	–	–		0.97 (0.71;1.32)	0.84 (0.59;1.20)	1.03 (0.87;1.22)			1.02 (0.85;1.23)
4 (wildtype; wildtype; variant)													
0 copies	87% (750)	91% (685)	1 (ref.)	1 (ref.)	89% (688)	88% (207)		1 (ref.)	1 (ref.)	1 (ref.)			1 (ref.)
1 copy	13% (108)	9% (68)	0.74 (0.54;1.03)	0.77 (0.54;1.11)	10% (79)	11% (26)		1.11 (0.68;1.81)	1.07 (0.59;1.95)	0.82 (0.62;1.08)			0.83 (0.61;1.13)
2 copies	0% (3)	0% (2)	0.91 (0.14,5.85)	0.78 (0.12;5.16)	0% (3)	0% (1)		0.99 (0.10;9.91)	0.80 (0.07;9.27)	0.93 (0.21;4.02)			0.88 (0.20;3.94)
1 or 2 copies (dominant model)	–	–	0.75 (0.54;1.03)	0.77 (0.54;1.10)	–	–		1.10 (0.68;1.79)	1.06 (0.59;1.90)	0.82 (0.63;1.08)			0.84 (0.62;1.13)
**Haplotype combinations**													
** 11**	6% (52)	6% (42)	0.85 (0.54;1.36)	0.99 (0.59;1.64)	6% (44)	6% (13)		1.06 (0.50;2.23)	1.09 (0.47;2.55)	0.89 (0.60;1.33)		1.01 (0.65;1.56)	
12	20% (173)	19% (147)	0.91 (0.66;1.24)	0.89 (0.63;1.26)	19% (144)	22% (51)		1.29 (0.79;2.12)	1.31 (0.77;2.23)	0.98 (0.75;1.28)		1.00 (0.74;1.34)	
13	11% (100)	12% (92)	0.96 (0.67;1.38)	0.97 (0.65;1.45)	13% (104)	12% (28)		0.97 (0.55;1.70)	0.88 (0.47;1.63)	0.96 (0.71;1.30)		0.97 (0.69;1.36)	
14	3% (27)	2% (17)	0.72 (0.37;1.40)	0.79 (0.36;1.72)	3% (21)	3% (7)		1.11 (0.42;2.94)	0.70 (0.20;2.48)	0.80 (0.46;1.39)		0.78 (0.40;1.53)	
** 22**	19% (171)	22% (163)	1 (ref.)	1 (ref.)	20% (152)	17% (40)		1 (ref.)	1 (ref.)	1 (ref.)		1 (ref.)	
23	24% (212)	24% (184)	0.93 (0.69;1.25)	0.93 (0.67;1.30)	24% (183)	27% (62)		1.22 (0.76;1.96)	0.96 (0.56;1.67)	0.98 (0.76;1.27)		0.95 (0.72;1.26)	
24	6% (51)	4% (28)	0.61 (0.36;1.02)	0.59 (0.34;1.03)	5% (37)	5% (12)		1.17 (0.55;2.52)	1.26 (0.54;2.97)	0.72 (0.46;1.11)		0.70 (0.44;1.12)	
** 33**	7% (62)	8% (57)	1.01 (0.66;1.55)	0.99 (0.62;1.59)	8% (65)	6% (13)		0.82 (0.40;1.68)	0.76 (0.34;1.70)	0.97 (0.67;1.39)		0.98 (0.65;1.46)	
34	4% (35)	3% (23)	0.84 (0.47;1.50)	0.98 (0.52;1.82)	3% (22)	3% (7)		1.49 (0.57;3.85)	1.45 (0.51;4.16)	0.94 (0.57;1.56)		1.08 (0.63;1.85)	
** 44**	0% (3)	0% (2)	0.85 (0.13;5.58)	0.73 (0.11;4.93)	0% (3)	0% (1)		1.09 (0.11;11.2)	0.79 (0.07;9.51)	0.91 (0.21;3.96)		0.86 (0.19;3.90)	
Not defined	0% (1)	0% (0)	–	–	0% (1)	0% (0)		–	–	–		–	

Median follow up 7.2 years. Diet, Cancer and Health, Denmark, 1993–2003.

^1^^)^Age used as time axis. Adjusted for educational level, waist circumference, smoking status, alcohol consumption, hypertension, diabetes mellitus and hypercholesterolemia. Women also adjusted for menopausal status.

^2^^)^Also adjusted for sex.

Due to the difference in biology and prevalence of ACS between the sexes, we performed separate analyses in men and women. However, if results were similar for men and women, we also conducted combined-sex analyses. All analyses were performed using Stata version 11.2 (StataCorp. 2009. *Stata Statistical Software: Release 11*. College Station, TX: StataCorp LP).

## Results

Baseline characteristics of the participants are presented in Table 1. Compared to the subcohort, both male and female cases appeared on average older, drank less alcohol, and fewer were never smokers. Similarly, cases had a higher prevalence of hypertension, diabetes mellitus, and hypercholesterolemia at baseline. More cases were overweight or obese, and overall few were underweight according to the WHO classification.

Haplotypes were inferred manually, and we identified 4 haplotypes which described more than 99.9% of the genotype combinations (Table 2). The haplotypes were identical to previous findings in Caucasians [12]. All genotypes were in Hardy-Weinberg equilibrium (data not shown), and the minor allele frequencies were similar among men and women (Table 3). Haplotype 1 included the wildtype allele of all three SNPs (Table 2), but more participants were homozygote for haplotype 2 than haplotype 1 (Table 4). Thus, participants with haplotype combination 22 were used as reference group in the analyses.

We did not find any significant associations between the individual SNPs and incident ACS (Table 3) among men or women, and analyses of both sexes combined yielded very precise neutral estimates. Likewise, none of the individual haplotypes or haplotype combinations showed significant associations with ACS (Table 4).

When we investigated the combined effect of SNP haplotypes 1, 2 and, 3 and obesity (BMI, WC adjusted for HC, HC adjusted for WC) or the *NFKB1* -94 ATTG ins/del polymorphism, the results showed no indication that haplotype carrier status modified the effect of the investigated variable. Cross tabulations are presented in an online supplement (Table S1 and Table S2). The prevalence of haplotype 4 was low, and thus haplotype 4 was omitted from the interaction analyses.

## Discussion

In this large prospective study, we found no significant associations between individual SNPs or SNP haplotypes in the promoter region of the *IL1B* gene and incident ACS. Furthermore, we found no evidence that the investigated SNP haplotypes modified the effect of obesity on the risk of ACS. Confidence limits show that it is quite unlikely that a possible true association could have given rise to the estimates.

The biological effect of the investigated SNPs on *IL1B* expression [12] and release of IL-1B [7] is well established, and the lack of statistically significant associations in our study suggests that IL-1B may have a limited role in the development of ACS in men and women above the age of 50.

The potential for selection bias during follow-up was limited as the proportion lost to follow-up was very small. We achieved a high level of data ascertainment, as all cases were validated by direct review of medical records and data on anthropometric variables were collected by trained study technicians according to a standardized protocol. Due to the subtle nature of atherosclerosis, reverse causation is a possibility in the anthropometry analyses, but we have previously found similar associations for all anthropometric variables when conducting analyses in participants who experienced an event within the first two years of follow-up and in participants who experienced an event after more than two years of follow-up [26].

There are several risk factors for ACS, and though these covariates may not be causally correlated with the genetic exposure variables, they may still be unevenly distributed among exposed and unexposed due to chance. However, adjustment for confounding had only limited effect on the associations suggesting that residual confounding was unlikely to play a major role for our findings.

The interpretation of the analyses of individual SNPs has been extensively discussed in the literature [12], [27], but it still seems reasonable to investigate functional SNPs in relevant genes if the minor allele frequency is high. In case of a significant association, further investigation may reveal whether the association is due to an isolated effect of the investigated SNP or a haplotype marked by the SNP. In the present study, we investigated 3 SNPs in the same promoter region, and, as expected, the SNPs were tightly linked, and thus only four haplotypes included more than 99.9% of the genotypes. The C-3737T and G-1464C SNPs were each present on only one haplotype which explains the almost identical results concerning C-3737T and haplotype 2 and G-1464C and haplotype 3, respectively (Table 3 and 4). Likewise, it is noteworthy that the variant C allele of the G-1464C SNP was only present in haplotype 3 which also included the variant C allele of the T-31C SNP, and thus, the isolated associations for the G-1464C SNP could not be examined in our cohort. The T-31C SNP was present on both haplotype 3 and 4, and thus, homozygote variant -31C carriers may be either homozygote for haplotype 3 or haplotype 4 or be heterozygote and carry both haplotype 3 and 4. This demonstrates a key problem in the analysis of individual SNPs, as analysis of the T-31C SNP will be affected by the presence of G-1464C among some, but not all, of the variant -31C carriers.

The identified haplotypes and their distribution were similar to the results of Chen et al [12], who found that haplotype 1 (all wildtype alleles) had the lowest transcriptional activity in response to LPS. Both haplotype 2 and 3 showed higher activity than haplotype 1, but their activities were not significantly different, whereas haplotype 4 showed the highest activity (Table 2). However, Chen et al only investigated individual haplotypes and not haplotype combinations as they are present in human genome, and thus the combined effect of haplotypes, e.g. haplotype 1 on the maternal allele and haplotype 2 on the paternal allele, is not clear. We expected a stepwise higher risk of ACS from haplotype combination 11 (lowest *IL1B* activity) through haplotype combinations 22 and 33 up to haplotype combination 44 (highest *IL1B* activity) if *IL1B* activity was associated with ACS, but this could not be demonstrated (Table 4). The associations for the discordant haplotype combinations could not be predicted, and we found no statistically significant associations for any of them.

Iacoviello et al found that the variant allele of the C-511T SNP, and relatively low IL-1B, was protective against myocardial infarction at young age [7]. Based on the same study population, Latella et al observed a similar reduced risk of myocardial infarction at young age for participants carrying haplotypes containing the variant -511T SNP [28]. In Danes, the C-511T SNP is in complete linkage disequilibrium with C-31T [14], and thus both haplotype 3 and 4 in our study must contain the variant allele of the C-511T SNP. This is in line with the fact that the inferred haplotypes in the study by Latella et al were identical to the haplotypes determined in our study, given that the C-31T and the C-511T SNPs are in complete linkage disequilibrium. However, we found no evidence of a consistent significant association between incident ACS and either the T-31C SNP or haplotype 3 or 4 (Table 3 and Table 4). This could be due to a “healthy participant effect” since the participants in our study were at least 50 years of age at inclusion, and we excluded all participants with prevalent ACS. Participants who had “survived” until inclusion without sustaining ACS might have had a reduced susceptibility to the *IL1B* SNPs.

In conclusion, genetic variation in the promoter region of the *IL1B* may not be associated with incident ACS in men or women above the age of 50.

## Supporting Information

Table S1 **Hazard ratios (HR) with 95% confidence interval in brackets for the association between baseline anthropometric measures, IL1B SNP haplotypes and incident acute coronary syndrome.** Median follow up 7.2 years. Diet, Cancer and Health, Denmark, 1993-2003.
(XLS)

Table S2 **Relative excess risk due to interaction (RERI) with 95% confidence interval in brackets for the association between baseline anthropometric measures, IL1B SNP haplotypes and incident acute coronary syndrome.** Median follow up 7.2 years. Diet, Cancer and Health, Denmark, 1993–2003.
(XLS)
